# Enhancing Single-Plane Fluoroscopy: A Self-Calibrating Bundle Adjustment for Distortion Modeling

**DOI:** 10.3390/diagnostics14050567

**Published:** 2024-03-06

**Authors:** Jackson Cooper, Jacky C. K. Chow, Derek Lichti

**Affiliations:** 1 Department of Geomatics Engineering, University of Calgary, Calgary, AB T2N 1N4, Canada; 2 Department of Radiology, Cumming School of Medicine, University of Calgary, Calgary, AB T2N 2T9, Canada

**Keywords:** single-plane fluoroscopy, geometric distortion correction, 3D point reconstruction, quantitative measurement, self-calibration

## Abstract

Single-plane fluoroscopy systems with image intensifiers remain commonly employed in a clinical setting. The imagery they capture is vulnerable to several types of geometric distortions introduced by the system’s components and their assembly as well as interactions with the local and global magnetic fields. In this study, the application of a self-calibrating bundle adjustment is investigated as a method to correct geometric distortions in single-plane fluoroscopic imaging systems. The resulting calibrated imagery is then applied in the quantitative analysis of diaphragmatic motion and potential diagnostic applications to hemidiaphragm paralysis. The calibrated imagery is further explored and discussed in its potential impact on areas of surgical navigation. This work was accomplished through the application of a controlled experiment with three separate Philips Easy Diagnost R/F Systems. A highly redundant (~2500 to 3500 degrees-of-freedom) and geometrically strong network of 18 to 22 images of a low-cost target field was collected. The target field comprised 121 pre-surveyed tantalum beads embedded on a 25.4 mm × 25.4 mm acrylic base plate. The modeling process resulted in the estimation of five to eight distortion coefficients, depending on the system. The addition of these terms resulted in 83–85% improvement in terms of image point precision (model fit) and 85–95% improvement in 3D object reconstruction accuracy after calibration. This study demonstrates significant potential in enhancing the accuracy and reliability of fluoroscopic imaging, thereby improving the overall quality and effectiveness of medical diagnostics and treatments.

## 1. Introduction

Single-plane fluoroscopy remains a valuable modality due to its real-time temporal resolution, positional flexibility, and potentially lower radiation dosage relative to other cross-sectional imaging systems [1] such as computed tomography (CT) scans. The advantages of fluoroscopy make it particularly suitable for minimally invasive image-guided interventions [2] where temporal resolution is paramount.

Fluoroscopy systems have traditionally involved the use of an image intensifier. However, newer-generation, solid-state systems have also become available. The latter benefits from significantly reduced distortion [3] compared to the former, with the former also requiring quality control programs to monitor inevitable changes caused from the intensifier [4]. Despite the benefits of newer-generation, solid-state systems, image intensifier systems are still widely adopted. This work therefore specifically focuses on fluoroscopy systems with image intensifiers, addressing the critical issue of their image quality.

The advent of high-precision robotic platforms has been effective in supporting patient outcomes in several interventions [5,6,7,8]. The accuracy of such robotic tools is closely linked with that of fluoroscopic imaging systems due to their use in the navigation and application of these tools. Consequently, enhanced image quality in pursuit of higher accuracy than the robotic system itself instills increased confidence in the performed intervention. A critical determinant of this accuracy is the understanding of a camera system’s internal characteristics, achieved through the estimation of its interior orientation parameters (IOPs). The IOPs comprise key elements of the “internal” geometry of an imaging system as well as distortion coefficients that characterize the departures from ideal imaging geometry.

Several types of distortion can significantly impact the system’s IOPs, resulting in image warping and decreased accuracy. This distortion can be particularly troublesome in applications where the quantification of imaging features is required for diagnosis [9] or anatomical navigation [10,11].

This distortion is composed of the following elements: radial, decentering, affinity, s-shaped sigmoidal, and local distortions. Radial distortion, characterized by a pincushion effect, emerges from non-planar projection through the imaging system optics (such as from the curved image intensifier) [10] or onto a non-planar sensor surface [12]. Decentering distortion can result from the misalignment of imaging system components [13]. Affinity distortion is typically associated with an unequal imaging sensor array on the x- and y-axes [12], comprising non-orthogonality in the resulting image due to several potential factors. Sigmoid distortion arises as a function of the system’s external orientation to the Earth’s magnetic field [14,15]. Conversely, local non-homogenous magnetic fields from surrounding equipment and the environment induce local distortion [11,16]. The five distortion effects all result in the displacement of the image points from their expected locations.

The estimation and correction of these distortions can be achieved using global polynomial estimation [16]. This approach fits a generalized polynomial across the fluoroscopy system’s orientation range. However, due to its generalized nature, it does not provide insights on the characteristics of the types of distortion modeled. Recent studies have also detailed the use of self-supervised methods for the accurate modeling of systematic distortion within fluoroscopy systems. This is accomplished through the integration of iterative maximum likelihood estimation and k-nearest-neighbor regression [17].

Alternatively, the rigorous self-calibrating bundle adjustment has been effectively used to estimate and rectify distortion within imagery captured from a dual-fluoroscopy system [18]. This method has been previously explored in the context of research lab systems. This study aims to explore the application of this method within the context of single-plane fluoroscopy in the estimation and correction of image distortion in systems used in outpatient radiology clinics and hospital settings.

The application of this method within the context of a clinical setting introduces the possibility of improved quantitative analysis and observation of anatomical and clinically relevant features. This form of calibration creates a set of common and calibrated imagery which can be used to establish new measurement baselines. This concept has been successfully explored within the context of diaphragm paralysis analysis [19].

## 2. Materials and Methods

Data from a target field were collected at two Canadian academic hospitals to determine three distinct fluoroscopy machines’ distortion profiles and calibration parameters. This study further provided quality control of the fluoroscopy systems through the analysis of accuracy for systems used clinically on a daily basis.

This was achieved by implementing a free-network, self-calibrating bundle adjustment [20], a rigorous and flexible calibration method. This method fundamentally relies on the principle of the collinearity condition, which postulates that a target—whether a patient or phantom—in 3-dimensional (3D) object space aligns in a straight (collinear) line with its 2-dimensional (2D) projection captured in image space.

This model can be expressed in terms of object point *i*, image point *j*, and imaging system *k,* in the following expression with respect to the *x-*, *y*-axes.

(1) $$x_{ijk}+\varepsilon_{x_{ijk}}=x_{Pk}-c_{k}\frac{U_{ij}}{W_{ij}}+∆x_{ijk}$$

(2) $$y_{ijk}+\varepsilon_{y_{ijk}}=y_{Pk}+c_{k}\frac{V_{ij}}{W_{ij}}+∆y_{ijk}$$

The terms on the left of Equations (1) and (2) include the coordinates of the observed image points ($x_{ijk},y_{ijk}$) in an image space coordinate system. The additive terms of ($\varepsilon_{x_{ijk}},\;\varepsilon_{y_{ijk}}$) represent the zero-mean white noise error to the observation. The right of the equation includes the imaging system’s principal point ($x_{Pk},\;y_{Pk}$) and principal distance ($c_{k}$). The geometric distortion correction coefficients of the imaging system are represented by ($∆x_{ijk},\;∆y_{ijk}$). It is worth noting that the principal point, principal distance, and geometric distortion correction coefficients all contribute to the overall image distortion between the expected and observed image points. The transformation between the object space coordinates to the image space coordinates is seen in the relational term multiplied by the principal distance. This relationship is typically between the coordinates in millimeters (mm) and pixels (pix), with the transformation defined in the following.

(3) $${\left(\begin{array}{c} U\\ V\\ W \end{array}\right)}_{ij}={M(\omega ,\phi ,\kappa )}_{j}\left(\begin{array}{c} X_{i}-X_{j}^{c}\\ Y_{i}-Y_{j}^{c}\\ Z_{i}-Z_{j}^{c} \end{array}\right)$$

In Equation (3), the difference between the object space coordinates ($X_{i},\;Y_{i},Z_{i}$) and the principal center coordinates ($X^{C},Y^{C},Z^{C}$) is taken. The coordinate difference is multiplied by the rotation sequence of $M_{j}=R_{3}(\kappa_{j})R_{2}(\phi_{j})R_{1}(\omega_{j})$, where (ω,ϕ,κ) are rotations with respect to the (X, Y, Z) primary, secondary, and tertiary axes. For additional information and robust implementation of the collinearity equation and self-calibrating bundle adjustment, please refer to additional resources [12,13,20].

We can estimate the parameters of this relationship using a weighted least-squares adjustment (LSA). The LSA can also estimate exterior orientation parameters (EOPs) which represent the orientation and position of the captured fluoroscopy imagery relative to the phantom/target of interest.

Moreover, the functional model can be augmented by incorporating empirically determined image distortion models to account for systematic errors introduced by the fluoroscopy system. These parameters are estimated within the LSA framework, offering a robust strategy to mitigate the impact of distortions in the imaging process.

A low-cost, custom-made target field was utilized as the calibration frame for this study. It comprises a 25.4 mm × 25.4 mm acrylic sheet embedded with 121 regularly spaced radiopaque and solid tantalum beads. The 3D coordinates of these beads were estimated approximately from the machine-drilled holes in the acrylic sheet, serving as initial values in the bundle adjustment. It is important to note that the beads were also surveyed with high accuracy to provide additional quality control and to assess accuracy, surpassing what is achievable via the self-calibration bundle adjustment for comparison. Such precision is not necessary in a clinical deployment, in order to keep costs low. Other radiopaque materials could be chosen to keep costs low, such as steel beads [18], which have been successfully utilized. The size of the radiopaque material is a balance between the density of points on the calibration frame and the visibility of the target in the imagery. The shape of the radiopaque material should be one in which it is easy to estimate the center of the object. A sphere is a strong choice, as ellipse fitting algorithms or centroid algorithms can be used to determine its position.

Fluoroscopic images were obtained using three distinct radiography/fluoroscopy rooms across two academic hospitals, each equipped with an image intensifier (Easy Diagnost, Phillips Medical Systems, Amsterdam, The Netherlands). These systems were chosen due to their clinical relevancy, as they are used for patient care on a daily basis. This further increases the opportunity for improving patient care in systems that are being actively utilized.

Fluoroscopic spot images and exposure images (lower and higher X-ray radiation, respectively) were captured. The acquired images were stored in a 512 × 512 8-bit digital format for subsequent processing.

For accurate distortion modeling of systematic errors within the imagery, it is imperative to have a highly redundant set of observations as part of the LSA. However, the LSA estimation process often yields a high level of correlation between the IOPs and EOPs, leading to poor numerical conditioning of the model. To de-correlate these parameters, an established approach to the network design of the captured imagery is required. This strategy should include highly convergent imagery, varying orientations (i.e., landscape and portrait imagery), a dense array of targets, and a wide distribution of targets across the field of view (FOV) [13].

**Figure 1:**
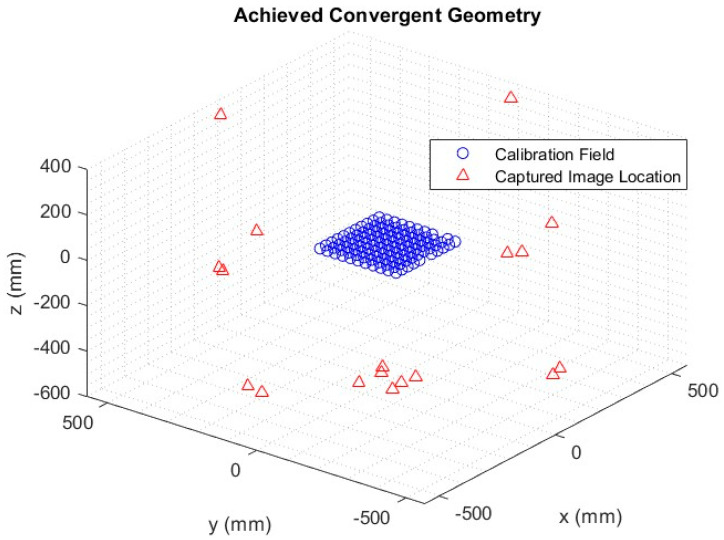
Single-fluoroscopic imaging system self-calibration network. Each image location (red triangle) around the calibration frame (blue circles) is shown.

These principles were incorporated into the network used for image capture (Figure 1) across all three fluoroscopy suites. The image capture process commenced with the target field positioned parallel to the image intensifier. This was followed by capturing multiple images with translation across the intensifier’s FOV. Subsequently, the target field was positioned at a 30° angle using a wooden stand and captured in one quadrant. The stand was then moved 90° to the next quadrant while the frame was also rotated by 90° within the stand, ensuring the same front face was maintained. This process was repeated for all four quadrants.

A distinctive feature of X-ray imaging systems, as compared to conventional camera systems, is their ability to “see through” or penetrate objects. We exploited this unique characteristic by rotating the target field 180° to its back face, with the rotations across all four quadrants repeated from this perspective. This allowed for highly convergent imagery across the entirety of the FOV. Alternatively, the data acquisition can be semi-automated by introducing a self-moving platform, such as a robotic arm or turn-table [18]; however, it would increase the operational cost.

**Figure 2:**
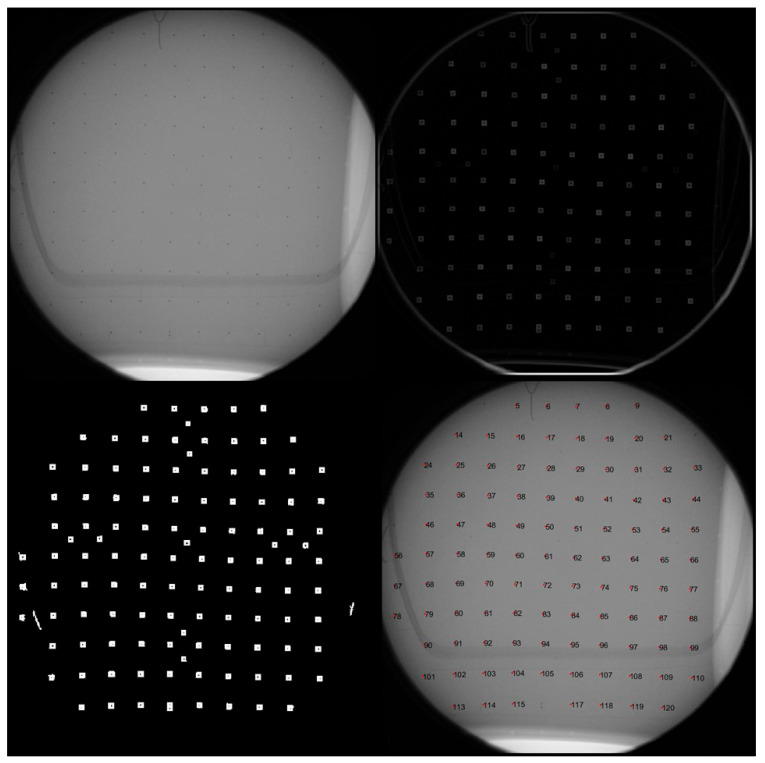
The target labeling process began with an example of captured imagery (**top left**) followed by morphological enhancement (**top right**). Extracted binary labels (**bottom left**) were then semi-automatically matched to image point labels through user selection. The centroids of the target field (**bottom right**) could then be extracted from the imagery. Note the significant curvilinear distortions visible in the image of the square target grid.

The 2D image coordinates of the tantalum beads were identified and labeled from the captured imagery using a custom-developed MATLAB program (MathWorks Inc., Version R2021a, Natick, MA, USA). In the captured imagery, the beads appeared only several pixels wide due to the small image format size and its required FOV. This necessitated morphological differencing to enhance the available detection area [21], with the resulting targets subsequently thresholded and converted into binary labels. The binary labels provide vertices that can be used to construct a polygon, with the resulting centroid equaling the average geometric center of the shape (Figure 2). The resulting targets were semi-automatically labeled based on their sequence, requiring minimal manual labeling and intervention (Figure 2). Labels were based on a predefined numeric identifier, which allowed for associating their 2D image coordinates with the corresponding 3D object coordinates.

The total statistics of the captured image point are summarized in the following table (Table 1).

The calibration model concurrently estimates all parameters, including IOPs, EOPs, and object space coordinates, within a single application of the LSA. The distortion parameters representing several types of distortion can all be modeled at once, as well. However, this can lead to overparameterization and poor determination of distortion correction coefficients, which necessitates an iterative and interactive approach. This can be conducted through statistical and graphical analyses, ensuring that each distortion parameter introduced in the model contributes significantly to the overall solution [13] and the model complexity minimizes the bias–variance tradeoff.

To assess the resulting accuracy, detached from the fitted model, an independent assessment was performed using a separate set of images (i.e., test dataset). This is accomplished with a limited image set with and without correction parameters applied. The external standard against which these results were compared is the independently surveyed target frame, which was captured with additional images. This approach allows for the evaluation of the effectiveness of the applied corrections effectively serving as a comparison to an external standard.

**Figure 3:**
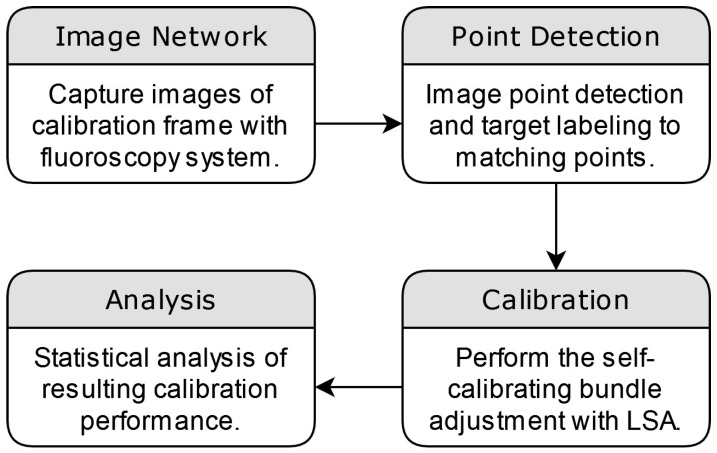
The overall methodology of the application of the self-calibrating bundle adjustment can be seen to begin with image capture and image point processing. The procedure is followed by the application of the calibration and subsequent analysis to determine the effectiveness of the applied correction.

As this study’s primary focus was on the geometric distortion of the imagery, the statistical methods chosen were specifically designed to evaluate the geometric differences between the estimated points derived from the calibration process and the pre-surveyed coordinates of the calibration frame. These statistics were further calculated to evaluate the estimated overall model quality. This included the root mean square error (RMSE) of the image residuals, which signifies overall model fit; 3D reconstruction accuracy through RMSE between the estimated and surveyed distance between coordinates; and the standard deviation of the estimated correction parameters, an indicator of the significance of the applied correction. The overall methodology from image capture to analysis is summarized in Figure 3.

## 3. Results

All evaluated fluoroscopy systems were similar regarding total captured imagery, the relative orientation of imagery, and the number of targets detected, as described in Table 1. All systems were evaluated with a highly redundant and well-conditioned functional model. This functional model consisted of five distortion parameters for system F1, eight for C1, and five for C2. These comprise the distortion categories of radial, decentering, affinity, s-shaped sigmoidal, and local distortions. Each resulting functional model was statistically significant with a standard deviation at least an order of magnitude smaller than the parameters.

Functional models were developed using both fluoroscopic spot imagery and exposure imagery. No differences were found with models developed using either form of the captured imagery.

The captured image point observations can be first evaluated through the RMSE of their residuals (observation error estimates) to quantify their fit to the functional model. The imaging systems were evaluated with pre- and post-application of distortion parameters (Table 2). All systems achieved a similar level of improvement, between 83 and 85%. The estimated variance factor (quadratic form of the residuals) can be further evaluated between pre- and post-application of estimated distortion parameters to provide an indication of the overall fit of the stochastic model to the observed image points. The imaging systems experienced an average enhancement of ~98% (Table 2), indicating effective modeling within the stochastic framework through the incorporation of distortion parameters.

**Figure 4:**
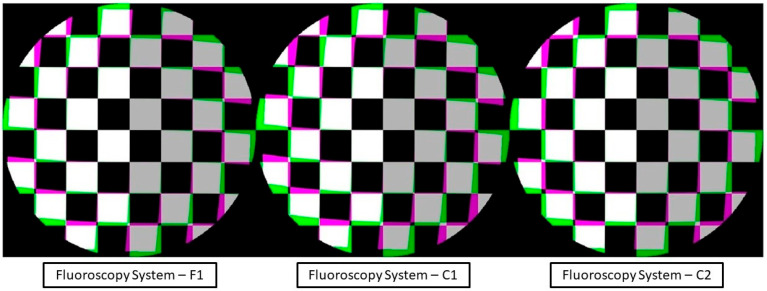
The virtual image of a checkerboard differenced with the distortion profile map for each fluoroscopic system. The distortion for all imagery can be seen to increase radially from the center, with the greatest magnitude of distortion towards the image edges. The largest distortion parameter magnitude for system F1 is affinity distortion, that of system C1 is decentering distortion closely followed by local distortion, and that of system C2 is affinity distortion.

The distortion profile could then be displayed in conjunction with the corresponding correction applied to the imagery. The estimated distortion parameters from the self-calibrating bundle adjustment were calculated and mapped to each pixel within the captured imagery. This in effect corrected the distortion field of the imagery. The distortion field could be further visualized by its application to a regularly spaced virtual image of a checkerboard. The resulting image (Figure 4) displays the difference in the checkerboard to its mapped distortion profile.

**Figure 5:**
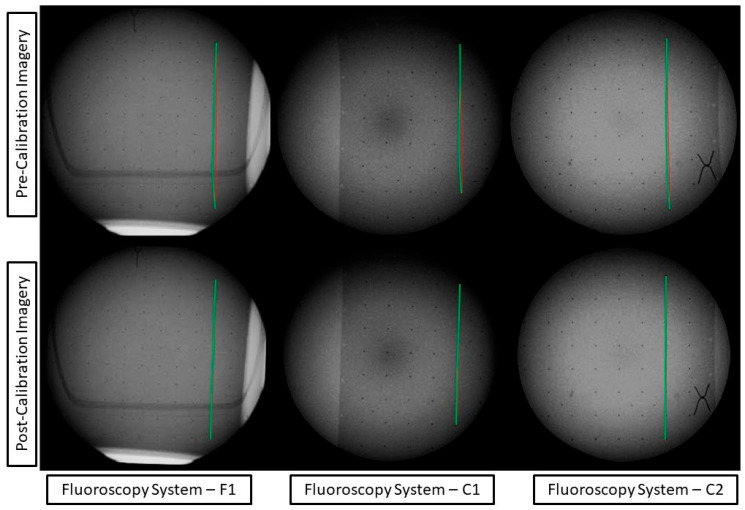
Imagery captured across fluoroscopy systems pre-correction and post-correction using distortion model parameters. Imagery is annotated with the curve of the tantalum beads represented by the green curve and an idealized straight line of the beads with a red line.

The distortion correction models can be applied to the captured imagery of the calibration frame to visualize the pre- and post-distortion correction (Figure 5).

The imaging systems’ 3D reconstruction accuracy was evaluated with pre- and post-application of distortion parameters (Table 3). Two independent accuracy assessments (with and without the modeled distortion parameters) were used to determine the overall model improvement in the fluoroscopy imaging systems. A subset of test images from each fluoroscopy system underwent the application of the bundle adjustment to estimate the 3D coordinates of the tantalum beads. Distances were subsequently calculated between the estimated coordinates and compared to the distances of the pre-surveyed ground truth coordinates. The residuals were then evaluated through their mean, standard deviation, and RMSE. The resulting improvement ranged from 83 to 97% for the test dataset (Table 3).

## 4. Diagnostic Application

The use of quantitative measurements in medical imaging can significantly enhance the accuracy and efficiency of diagnostics and surgical planning, offering a more precise and data-driven approach to patient care. Different quantitative methods have been successfully developed and have seen various levels of clinical adoption. One challenging diagnosis area that could benefit from quantitative measurements is detecting paradoxical diaphragm motion and diagnosing hemidiaphragm paralysis [19].

The gold-standard method for diagnosis is the fluoroscopic sniff test. The motion of the diaphragm is analyzed following inspiration to evaluate the presence of potential abnormalities in muscular contraction. However, the paradoxical motion between hemidiaphragms can be subtle and occur quickly [19]. The extraction of the diaphragm surface and temporal motion from fluoroscopic imagery can provide an objective measure of the statistical significance of any potential paradoxical motion. This can be an essential indicator in the prognosis and selection of treatment plans such as diaphragm plication [19].

The geometric calibration of fluoroscopic imagery provides improved confidence in the statistics and detection of diaphragm motion. This is important, as potential abnormalities can be missed due to sensitive geometry.

To demonstrate this, fluoroscopic imagery from a patient with chronic left hemidiaphragm paralysis [19] was calibrated to observe the changes in movement.

**Figure 6:**
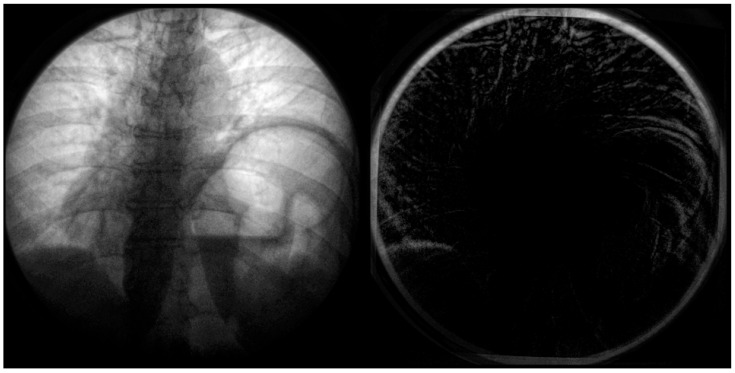
Imagery captured from a fluoroscopic sniff test at peak expiration from a case study of chronic left hemidiaphragm elevation [19]. A frame from the video fluoroscopy was captured pre-application of calibration (**left**). The geometric distortion coefficient and resulting calibration was applied to the imagery and differenced (**right**). Note the significant difference in the top of the diaphragm in the left of the image (**right**) between calibrated and non-calibrated imagery.

**Figure 7:**
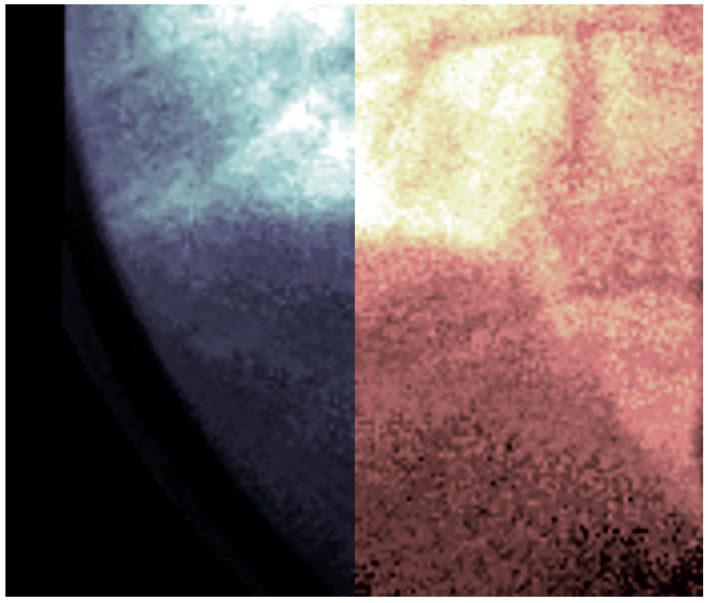
Fluoroscopic spot image of the right hemidiaphragm from a fluoroscopic sniff test at peak expiration from a case study of chronic left hemidiaphragm elevation [19]. Calibrated imagery (**right**) and non-calibrated imagery (**left**) are compared. The primary shadow along the centerline represents the diaphragm’s surface. The difference in geometric position between calibrated and non-calibrated imagery can be noted.

In the below Figure 6, the distortion is most significant radially from the center. This distortion appears primarily on anatomical features such as the ribcage, sternum, and diaphragm (Figure 7). This could significantly impact quantitative assessment if these features were used as landmarks. A side-by-side comparison can be used at the point of the diaphragm to visualize the difference between the calibrated and non-calibrated imagery.

To illustrate the difference between the calibrated and non-calibrated imagery across the range of motion of the diaphragm during normal inspiration, a volumetric approach to image analysis can be taken. The video fluoroscopy imagery can be stacked on the *z*-axis to form a volume. This volume can subsequently be sliced at a point near the diaphragm along the *x*-axis to visualize the contraction and relaxation of the muscle.

**Figure 8:**
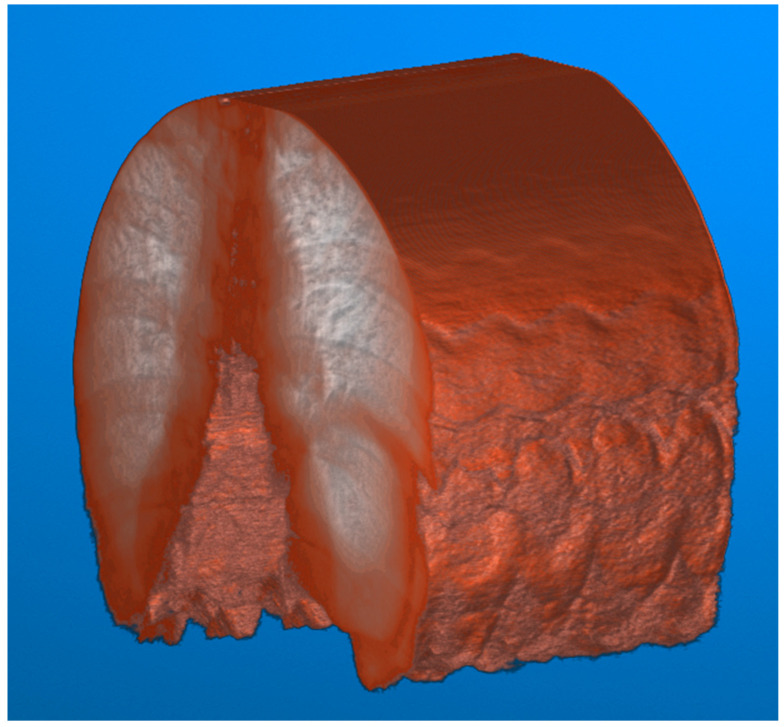
Video fluoroscopy from a case study of chronic left hemidiaphragm elevation [19] during normal inspiration. The resulting video imagery is stacked along the *z*-axis for a total of 229 frames of images. The image volume can then be visualized, with contrast adjustments applied for improved visualization. The movement of the lungs caused by the diaphragm can subsequently be visualized.

This visualization (Figure 8) results in a series of troughs representing the inspiration and contraction of the diaphragm and peaks representing the relaxation of the diaphragm. Each video frame of the video fluoroscopy can subsequently be calibrated to visualize the effect of distortion across the range of motion of the diaphragm.

**Figure 9:**
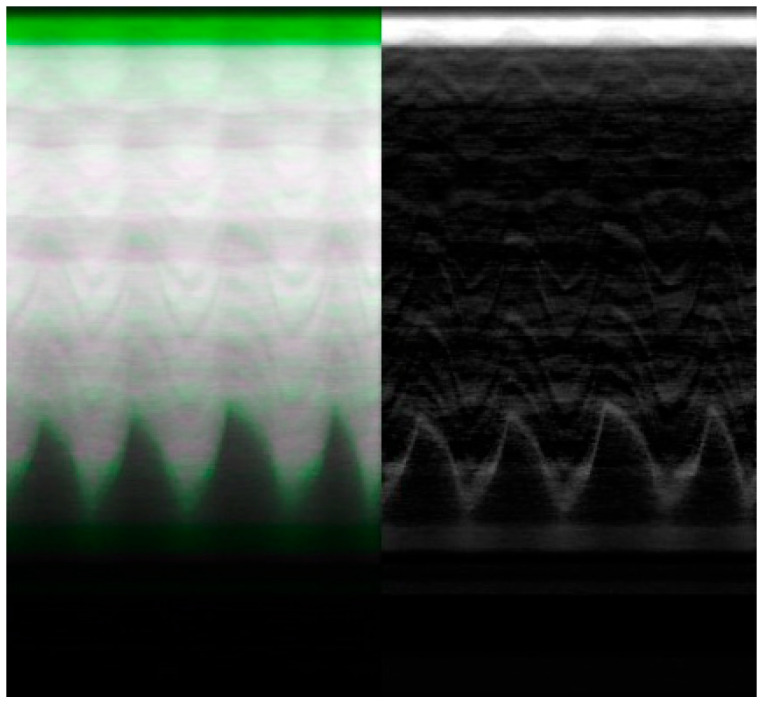
Video fluoroscopy from a case study of chronic left hemidiaphragm elevation [19] during normal inspiration of the right hemidiaphragm. The image volume is sliced along the y–z plane at a point near the diaphragm to visualize its movement. The geometric distortion coefficients were applied as a calibration to each frame within the video fluoroscopy. The resulting imagery is superimposed (**left**) and differenced (**right**). The distortion is most evident at peaks and troughs corresponding to the relaxation and contraction of the diaphragm, respectively.

The resulting imagery (Figure 9) demonstrates a potential expected difference between calibrated and non-calibrated imagery for the analysis of diaphragm motion. The distortion varies based on the movement of the diaphragm, with maximum distortion found at the contraction and relaxation of the diaphragm. The imagery (Figure 9) showcases the effects of distortion represented as the green overlay (left) and gray (right). This distortion can result in a difference of 10 pixels or more between calibrated and non-calibrated imagery.

## 5. Discussion

To a radiologist or surgeon, the visual difference in Figure 4 and Figure 5 may not be of utmost relevancy for routine diagnostic examination or interventions because our brains can automatically compensate for small distortions during interpretation. However, from a quantitative standpoint, the potential benefits are magnified. In quantitative fluoroscopy applications, such considerable distortion could lead to potential measurement inaccuracies of various anatomical features. This effect is further pronounced in applications where the imaging target spans the full FOV where the increased distortion lays. This is a crucial factor to consider when precise measurement is paramount, for example, in identifying and monitoring disease progression [9] or in planning and navigating for surgical intervention [10,11].

Specifically, in terms of 3D point reconstruction, the data underscore a consistent and marked improvement in object space accuracy. Before calibration, the 3D point accuracy as measured by RMSE across systems was, on average, 7.91 mm. Post-calibration, this error metric witnessed a sharp decline, registering an average of 0.68 mm. This denotes an average enhancement of approximately 91% across the three systems. This corrected distortion can be seen evidently across Figure 4 and Figure 5, with increasing distortion seen radially from the center.

The calibration performed has the potential to be incorporated into existing quality assurance programs and workflows when the fluoroscopy system is being used outside of regular clinical diagnostics. The materials required to perform the calibration are low-cost, as the imaging phantom only requires acrylic sheets and embedded radiopaque beads. The time requirement for image capture is minimal, taking a maximum of twenty minutes to carefully capture the required image network per fluoroscopy machine. This time can be further reduced if a device such as a turn-table or other device optimizes the motion of the calibration frame. Following image network capture, the calibration analysis is performed fully through software, allowing for remote analysis by a technician. Once the calibration parameters are determined, the imagery can be corrected in real time if integrated into manufacturers’ existing software architecture.

Calibrating the system appropriately can also potentially enhance the FOV in the captured images. Post-calibration, the decrease in distortion near the edges of the imagery could eliminate the need to limit the FOV to a circular shape. Instead, it becomes potentially feasible to expand the collimation cone mask to nearly the full size of the rectangular detector. This enhancement could be particularly beneficial for patients who face challenges in positioning during image capture, as it allows for greater flexibility and coverage in imaging.

In the realm of robot-assisted endovascular surgery, calibration holds paramount importance, given the intricate interdependence between the precision of robotic tools and fluoroscopy imaging systems [5,6,7,8]. Any distortion or inaccuracy in fluoroscopy can significantly skew measurements used in and before surgical procedures. The quantitative improvements in both 3D point precision and external orientation ensure that the utilized imagery is not compromised or the limiting factor is in precision, ultimately bolstering confidence in surgical interventions.

This is particularly valuable in endovascular procedures such as transcatheter aortic valve implantation (TAVI) where fluoroscopy is used extensively [22]. Fluoroscopy plays a critical role in guiding implantation and the confirmation of final valve sizing [23,24]. Through image fusion with preoperative imaging, fluoroscopic imaging angles can be determined, reducing the use of repeat contrast media [25].

The calibration of fluoroscopic Imagery was further demonstrated in its impact on the quantitative diagnosis of hemidiaphragm paralysis and detection of paradoxical diaphragm motion [19]. Specifically, significant differences between calibrated and non-calibrated imagery can be observed in the position of the diaphragm at peak contraction and relaxation as visualized by the peaks and troughs of the periodic motion of regular breathing (Figure 9). This difference could potentially skew the significance and confidence in any paradoxical motion.

The study, as described, is limited to the exploration of single-plane fluoroscopic systems. Traditionally in surgical intervention, C-Arm-based systems are preferred. Due to the prevalence of distortion that is tied to its spatial location within the magnetic field, it is to be seen to what degree of movement of the C-Arm within a surgical setting would affect the resulting distortion profile. This future suggests research in exploring the possibility of a transformation profile to correct for differences in the distortion field across the movement. The calibration as described further has the potential to be extended to other radiation-emitting imaging technologies. In particular, portable C-Arm imaging systems and cone-beam CT are strong candidates for the application of this calibration methodology. Furthermore, future investigations into the application of quantitative surgical navigation would be useful.

Several methods have been successfully implemented for the geometric calibration of fluoroscopy imaging systems, including global polynomial estimation [16] and maximum likelihood estimation [17]. These methods each have their relative strengths and weaknesses; the self-calibrating bundle adjustment method presented in this study also faces unique challenges. Specifically, our method requires additional setup and effort in capturing the image network to obtain the robust initial estimations of EOPs necessary for the model. Additionally, it necessitates precise image point detection of the calibration frame. However, these challenges can be mitigated with further research and development. There is also substantial value in future studies focusing on the quantitative and qualitative comparison of different geometric calibration procedures and algorithms. Ultimately, there is a need to provide a clearer understanding of different methods’ comparative advantages and limitations.

As computer vision systems become more reliant on artificial intelligence (AI), as it provides the possibility for segment-anything models (SAMs), how the level of geometric distortion affects these algorithms is to be seen. It has been reported that an increase in several noise profiles affects the labeling from convolutional neural networks (CNNs) [26], and geometric distortion can potentially be thought of as another systematic distortion that varies based on the system’s unique implementation characteristics. This study’s authors propose incorporating geometric distortion into the training of foundational CNN models. This approach, widely recognized as image augmentation [26], plays a critical role in both comprehensive end-to-end training and in the process of domain transfer learning for CNNs. Specifically, by enriching the training dataset of a foundational model with diverse distortion profiles that mimic a variety of fluoroscopy systems, the model’s robustness and its ability to generalize across different real-world scenarios can potentially be significantly enhanced. This strategy points to a promising research direction, emphasizing the potential benefits of integrating geometric distortion profiles into the process of augmenting training datasets for future image-based models.

## 6. Conclusions

The implementation of a self-calibrating bundle adjustment resulted in substantial enhancements in geometric accuracy for fluoroscopic imagery. These improvements were seen across 3D point reconstruction accuracy (Table 3), as well as the image measurement precision and overall model fit (Table 2), with the magnitude of improvement similar across all systems. These improvements were achieved through the application of distortion parameters estimated through the self-calibrating bundle adjustment. These estimated parameters have the benefit of being tied directly to physical phenomena [4,11,12,13,14,15], allowing for improved understanding of system performance. This had the further benefit of being a potential indicator for unexpected system errors unapparent in traditional calibration examinations. Differences ultimately existed between the applied distortion parameters between the imaging systems, potentially explained by the variations in the amount of wear and tear between the systems. These discrepancies can also potentially be explained by the variations in the ambient magnetic fields where the fluoroscopy machines are located. Ultimately, these differences can be investigated through a non-destructive method that does not void the manufacturers’ warranty of a specific system with the use of the self-calibrating bundle adjustment. The opportunity for improved geometric imagery can contribute to the fields of automatic diaphragm motion analysis and potential use in improved accuracy for TAVI angle of implantation.

## Figures and Tables

**Table 1:** Total captured imagery across fluoroscopy systems and total degrees of freedom.

Fluoroscopy System	Number of Images	Number of Observed Image Points	Degrees of Freedom
F1—Phillips Easy Diagnost—Hospital 1	22	1998	3512
C1—Phillips Easy Diagnost—Hospital 2	18	1505	2541
C2—Phillips Easy Diagnost—Hospital 2	18	1497	2528

**Table 2:** Model fit of observations in x, y image space axes through RMSE pre-correction and post-correction using distortion model parameters.

Fluoroscopy System	Self-Calibrating Bundle Adjustment	RMS x (Pixels)	RMS y (Pixels)	Estimated Variance Factor
F1	Pre-correction	1.27	1.25	35.61
	Post-correction	0.22	0.22	1.08
	Percent improvement (%)	83	83	97
C1	Pre-correction	1.67	1.68	65.34
	Post-correction	0.25	0.29	1.66
	Percent improvement (%)	85	83	98
C2	Pre-correction	1.39	1.40	45.29
	Post-correction	0.21	0.22	1.09
	Percent improvement (%)	85	84	98

**Table 3:** Accuracy assessment using test imagery and estimated distances between target field coordinates. The mean residual between known and estimated coordinates, standard deviation, and RMSE improvements to pre-correction and post-correction using distortion model parameters.

Fluoroscopy System	Self-Calibrating Bundle Adjustment	Mean Residual (mm)	Standard Deviation (mm)	RMSE (mm)
F1	Pre-correction	−5.60	5.37	7.73
	Post-correction	−0.16	0.34	0.37
	Percent improvement (%)	97	94	95
C1	Pre-correction	−3.90	6.10	7.19
	Post-correction	−0.16	0.34	0.37
	Percent improvement (%)	96	94	95
C2	Pre-correction	−5.61	6.82	8.80
	Post-correction	−0.93	0.92	1.30
	Percent improvement (%)	83	87	85

## Data Availability

The data presented in this study are available on request from the corresponding author.
